# Impact of ALDH2 genotypes and alcohol consumption on age at first‐ever ischemic stroke: A cohort study in Taiwan

**DOI:** 10.1111/acer.70217

**Published:** 2025-12-01

**Authors:** Yueh‐Feng Sung, Jiunn‐Tay Lee, Chaur‐Jong Hu, Jiann‐Shing Jeng, Hung‐Yi Chiou, Giia‐Sheun Peng

**Affiliations:** ^1^ Department of Neurology, Tri‐Service General Hospital National Defense Medical University Taipei Taiwan; ^2^ Department of Neurology Taipei Medical University Hospital and Shuang Ho Hospital Taipei Taiwan; ^3^ Stroke Center and Department of Neurology National Taiwan University Hospital Taipei Taiwan; ^4^ School of Public Health, College of Public Health Taipei Medical University Taipei Taiwan; ^5^ Institute of Population Health Sciences National Health Research Institutes Miaoli Taiwan; ^6^ Division of Neurology, Department of Internal Medicine Taipei Veterans General Hospital, Taoyuan Branch Taoyuan City Taiwan

**Keywords:** alcohol consumption, *ALDH2* polymorphism, gene–environment interaction, ischemic stroke, stroke onset age

## Abstract

**Background:**

The ALDH2*2 allele, common in East Asians, impairs aldehyde metabolism and leads to acetaldehyde accumulation during alcohol consumption. Whether this variant interacts with alcohol use to influence the age at ischemic stroke onset remains unclear. We evaluated the associations between ALDH2*2 and alcohol consumption with age at stroke onset.

**Methods:**

We enrolled 930 patients (598 men and 332 women) with first‐ever ischemic stroke. Alcohol consumption was dichotomized as heavy (>45 g/day or >300 g/week of ethanol) versus nonheavy (abstinent or ≤45 g/day and ≤300 g/week). Because heavy drinking was rare in women, primary analyses focused on men. Participants were grouped by the *ALDH2* genotype (*1/*1 vs. *1/*2 or *2/*2) and drinking category. Clinical variables were compared using analysis of variance and chi‐squared tests. Multivariable linear regression, adjusted for smoking, assessed associations with age at stroke onset.

**Results:**

Among men, mean age at stroke onset differed significantly across genotype–drinking groups (*p* < 0.001). ALDH2*2 carriers with heavy drinking had the earliest onset (56.1 ± 9.6 years), whereas ALDH2*2 carriers with nonheavy drinking had the latest onset (63.6 ± 12.3 years). Relative to *1/*1 with nonheavy drinking, ALDH2*2 carriers with nonheavy drinking had later onset (*β* = 2.67 years, *p* = 0.023), while those with heavy drinking had earlier onset (*β* = −4.61 years, *p* = 0.048). In the overall cohort, male sex, smoking, and heavy drinking were independently associated with younger onset (*β* = −3.20, −2.57, and − 3.40 years; *p* = 0.001, 0.006, and 0.002, respectively).

**Conclusions:**

In East Asian men, heavy alcohol consumption among ALDH2*2 carriers is associated with earlier ischemic stroke onset, suggesting a gene–environment interaction. These findings support genotype‐informed lifestyle counseling for stroke prevention.

## Graphical abstract

In a Taiwanese cohort of 930 patients with first‐ever ischemic stroke, we examined whether *ALDH2* rs671 (ALDH2*2) and alcohol consumption interact to influence age at stroke onset. In sex‐stratified analyses, men carrying ALDH2*2 with heavy drinking had the earliest onset, whereas ALDH2*2 carriers with nonheavy drinking had the latest, consistent with a harmful gene–environment interplay. Associations persisted after multivariable adjustment and support genotype‐informed counseling to minimize alcohol exposure, potentially delaying stroke onset in East Asian populations.

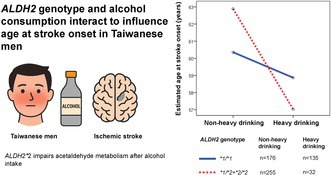

## INTRODUCTION

Ischemic stroke is a leading cause of death and long‐term disability worldwide, with a particularly high burden in Asian populations (GBD 2021 Stroke Risk Factor Collaborators, 2024). While traditional vascular risk factors—such as hypertension (HTN), diabetes mellitus (DM), and smoking—are important contributors, accumulating evidence suggests that genetic predisposition and gene–environment interactions play a critical role in modulating stroke susceptibility and phenotypic variation, including the age at onset (Shi et al., 2022).

Alcohol consumption has also been linked to stroke risk and timing. Large population‐based studies have shown that heavy or binge drinking increases the risk of ischemic stroke and may precipitate stroke onset at a younger age, whereas light‐to‐moderate intake shows inconsistent or nonlinear associations (Bazzano et al., 2007; Kadlecová et al., 2015).

A common functional polymorphism in the *aldehyde dehydrogenase 2* (*ALDH2*) gene, rs671 (ALDH2*2, Glu504Lys, also referred to as E487K in the mature protein), occurs almost exclusively in East Asian populations (Eng et al., 2007; Yin & Peng, 2007). The ALDH2*2 allele markedly reduces enzymatic activity, impairing acetaldehyde metabolism and leading to the accumulation of this toxic metabolite during alcohol breakdown (Lai et al., 2014; Peng & Yin, 2009). Carriers frequently experience alcohol‐flushing reactions and tend to consume less alcohol, although some continue to drink despite adverse effects (Peng et al., 2007).

Beyond alcohol intolerance, *ALDH2* polymorphism has been associated with several cardiovascular conditions, including coronary artery disease and ischemic stroke (Liu et al., 2024; Sun et al., 2017; Sung et al., 2016; Takeuchi et al., 2012). Our prior work in a large Taiwanese cohort identified the ALDH2*2/*2 genotype as an independent risk factor for ischemic stroke in men (Sung et al., 2016), suggesting underlying genetic vulnerability. However, direct evidence for an interaction between *ALDH2* genotype and alcohol exposure—particularly its impact on age at first‐ever ischemic stroke—remains limited.

In this study, we aimed to investigate whether alcohol consumption modifies the relationship between *ALDH2* genotype and age at first‐ever ischemic stroke. Given the high prevalence of ALDH2*2 in East Asians and its influence on drinking behavior (and related habits such as smoking), clarifying this gene–environment interaction may help identify subgroups at heightened risk for earlier onset stroke and inform genotype‐informed prevention strategies.

## MATERIALS AND METHODS

### Study design and participants

We included 930 patients with first‐ever ischemic stroke from four hospitals in northern Taiwan: Tri‐Service General Hospital, National Taiwan University Hospital, Taipei Medical University Hospital, and Taipei Medical University‐Shuang Ho Hospital. Ischemic stroke was confirmed by neuroimaging and clinical evaluation by board‐certified neurologists. A first‐ever stroke event was defined as the first occurrence (no history of stroke in prior medical records) of rapidly developing focal neurological deficits of presumed vascular pathogenesis lasting >24 h. The study was approved by the institutional review boards of all participating hospitals, and all patients provided written informed consent.

### Demographic features and clinical characteristics

Baseline data included age at stroke onset, sex, body mass index (BMI), HTN, DM, hypercholesterolemia (HC), coronary artery disease (CAD), and atrial fibrillation (AF), as defined in the Taiwan Stroke Registry (Hsieh et al., 2010). Smoking status was dichotomized as smokers (currently smoking or quit <2 years) and nonsmokers (never smoked or quit ≥2 years). Liver function impairment (LFI) was defined as aspartate transaminase or alanine transaminase level >40 IU/L. Renal function impairment (RFI) was defined as a serum creatinine level of >1.2 mg/dL. Alcohol consumption was dichotomized as heavy (>45 g/day or >300 g/week of ethanol) versus nonheavy (abstinent or ≤45 g/day and ≤300 g/week) (Yao et al., 2011).

### DNA collection and genotyping

Genomic DNA was extracted from peripheral blood samples using standard protocols. For a subset of early‐enrolled patients, *ALDH2* genotyping was performed using multiplex polymerase chain reaction–amplified product length polymorphism analysis, as described in our previous study (Sung et al., 2016). For the remaining patients, DNA concentration and purity were assessed using a NanoDrop One spectrophotometer (Thermo Fisher Scientific) (Wei et al., 2021). Genotyping was then conducted using the Affymetrix Axiom Genome‐Wide TWB 2.0 array (Thermo Fisher Scientific), which includes 752,921 probes targeting 686,463 SNPs, including rs671.

### Statistical analysis

Continuous variables were summarized as mean ± standard deviation (SD) and categorical variables as counts and percentages. Group comparisons were conducted using one‐way analysis of variance for continuous variables and chi‐squared tests for categorical variables, with Bonferroni correction for post hoc analyses when appropriate. Multivariable linear regression models were used to examine the associations between *ALDH2* genotype, alcohol consumption, and their combination with age at stroke onset. Dummy variables were created for genotype–drinking subgroups, and smoking was included as a covariate due to baseline differences. An interaction term (ALDH2*2 × alcohol) was tested in a separate model. Collinearity was assessed using variance inflation factors and tolerance values. To visualize the interaction effect, a general linear model was additionally employed to estimate marginal means adjusted for clinical covariates, and an interaction plot was generated accordingly. All statistical analyses were performed using SPSS version 19 (SPSS Inc., Chicago, IL, USA). A two‐sided *p* value <0.05 was considered statistically significant.

## RESULTS

### Baseline characteristics

Table 1 shows the clinical characteristics of patients with first‐ever ischemic stroke. Data are presented overall (total, *n* = 930) and stratified by *ALDH2* genotype and by sex (men, *n* = 598; women, *n* = 332). Across all *ALDH2* genotype groups, women consistently had a later stroke onset than men. Among men, significant differences were observed in age (*p* = 0.011) and BMI (*p* = 0.029) across the genotypes based on one‐way ANOVA. However, no pairwise BMI differences remained significant after Bonferroni correction. Male *1/*2 and *2/*2 carriers had a later age at stroke onset (62.7 ± 11.7 and 63.2 ± 14.4 years, respectively) than *1/*1 carriers (59.8 ± 11.8 years). In women, there were no significant age differences across genotypes (*p* = 0.372). Alcohol consumption differed markedly by genotype in men (*p* < 0.001), with 43.4%, 12.0%, and 8.1% of *1/*1, *1/*2, and *2/*2 carriers reporting heavy alcohol consumption, respectively. This genotype–drinking association was not significant in females due to their near absence of heavy drinking. In addition, smoking rates were similar across genotypes in both sexes but notably higher in men (~65%) than in women (8%–15%). There were no significant genotype‐related differences in the prevalence of vascular risk factors, such as HTN, DM, HC, CAD, AF, LFI, or RFI in either sex. Stroke subtype distributions as defined by the Trial of ORG 10172 in Acute Stroke Treatment (TOAST; ORG refers to danaparoid sodium, Orgaran) were comparable across genotypes for both men (*p* = 0.873) and women (*p* = 0.076). In the overall cohort (*n* = 930), multivariable regression analyses (Table 2) demonstrated that male sex, smoking, and heavy drinking were independently associated with younger age at stroke onset, whereas HTN, CAD, AF, and RFI were linked to later onset.

**TABLE 1:** Clinical characteristics of first‐ever ischemic stroke patients stratified by the *ALDH2* genotype (total, male, and female subgroups).

	*ALDH2* genotypes											
	Total				Male				Female			
	*1/*1	*1/*2	*2/*2	*p* value	*1/*1	*1/*2	*2/*2	*p* value	*1/*1	*1/*2	*2/*2	*p* value
Number of subjects	486	348	96		311	225	62		175	123	34	
Age, years	62.7 ± 12.8	63.8 ± 11.6	64.8 ± 14.8	0.252	59.9 ± 11.8	62.7 ± 11.7	63.2 ± 14.4	0.011	67.9 ± 12.9	65.8 ± 11.2	67.5 ± 15.4	0.372
BMI	25.5 ± 3.8	25.0 ± 3.9	25.0 ± 5.2	0.228	25.6 ± 3.5	25.0 ± 3.4	24.3 ± 3.8	0.029^†^	25.3 ± 4.3	25.0 ± 4.6	26.1 ± 7.0	0.516
HTN	358 (73.7)	249 (71.6)	66 (68.8)	0.562	232 (74.6)	160 (71.1)	38 (61.3)	0.098	126 (72.0)	89 (72.4)	28 (82.4)	0.444
DM	188 (38.7)	137 (39.4)	44 (45.8)	0.420	123 (39.5)	78 (34.7)	28 (45.2)	0.260	65 (37.1)	59 (48.0)	16 (47.1)	0.146
HC	282 (58.0)	197 (56.6)	50 (52.1)	0.557	176 (56.6)	125 (55.6)	29 (46.8)	0.362	106 (60.6)	72 (58.5)	21 (61.8)	0.915
CAD	46 (9.5)	29 (8.3)	11 (11.5)	0.627	29 (9.3)	19 (8.4)	6 (9.7)	0.924	17 (9.7)	10 (8.1)	5 (14.7)	0.516
AF	62 (12.8)	27 (7.8)	11 (11.5)	0.069	28 (9.0)	14 (6.2)	5 (8.1)	0.497	34 (19.4)	13 (10.6)	6 (17.6)	0.116
LFI	63 (13.0)	39 (11.2)	16 (16.7)	0.350	41 (13.2)	26 (11.6)	12 (19.4)	0.276	22 (12.6)	13 (10.6)	4 (11.8)	0.870
RFI	84 (17.3)	63 (18.1)	21 (21.9)	0.570	60 (19.3)	49 (21.8)	14 (22.6)	0.717	24 (13.8)	14 (11.4)	7 (20.6)	0.380
Smoking	219 (45.1)	155 (44.5)	45 (46.9)	0.920	203 (65.3)	145 (64.4)	40 (64.5)	0.979	16 (9.1)	10 (8.1)	5 (14.7)	0.502
Heavy drinking	141 (29.0)	28 (8.0)	5 (5.2)	<0.001	135 (43.4)	27 (12.0)	5 (8.1)	<0.001	6 (3.4)	1 (0.8)	0 (0)	0.201
TOAST												
Large vessel	167 (34.4)	102 (29.3)	27 (28.1)	0.350	102 (32.8)	64 (28.4)	21 (39.9)	0.873	65 (37.1)	38 (30.9)	6 (17.6)	0.076
Cardioembolism	54 (11.1)	25 (7.2)	10 (10.4)		25 (8.0)	16 (7.1)	4 (6.5)		29 (16.6)	9 (7.3)	6 (17.6)	
Small vessel	157 (32.3)	131 (37.6)	35 (36.5)		110 (35.4)	87 (38.7)	20 (32.3)		47 (26.9)	44 (35.8)	15 (44.1)	
Other determined	20 (4.1)	18 (5.2)	6 (6.3)		13 (4.2)	10 (4.4)	5 (8.1)		7 (4.0)	8 (6.5)	1 (2.9)	
Undetermined	88 (18.1)	72 (20.7)	18 (18.8)		61 (19.6)	48 (21.3)	12 (19.4)		27 (15.4)	24 (19.5)	6 (17.6)	

*Note*: Values for age and BMI are expressed as mean ± SD. Numbers in parentheses are percentages. Statistically significant differences were determined using the chi‐squared test for categorical variables and one‐way analysis of variance for continuous variables between different groups. Heavy drinking was defined as >45 g/day or >300 g/week of ethanol; nonheavy = abstinent or ≤45 g/day and ≤300 g/week.

Abbreviations: AF, atrial fibrillation; BMI, body mass index; CAD, coronary artery disease; DM, diabetes mellitus; HC, hypercholesterolemia; HTN, hypertension; LFI, liver function impairment (GOT > 40 IU/L or GPT > 40 IU/L); RFI, renal function impairment (creatinine > 1.2 mg/dL); TOAST, trial of ORG 10172 in acute stroke treatment.

^†^ *p* value from one‐way ANOVA; Bonferroni‐adjusted pairwise comparisons were not significant (all adjusted *p* > 0.05).

**TABLE 2:** Multivariable linear regression analyses of age at stroke onset in the overall cohort (total 930 patients, male/female 598/332).

Model 1	*β* (years), 95% CI	*p* value	Model 2	*β* (years), 95% CI	*p* value
Male (vs. Female)	−3.51 (−5.47 to −1.56)	<0.001	Male (vs. Female)	−3.20 (−5.13 to −1.26)	0.001
Smoking (yes vs. no)	−2.22 (−4.11 to −0.28)	0.024	Smoking (yes vs. no)	−2.57 (−4.44 to −0.70)	0.006
Heavy drinking (yes vs. no)	−4.12 (−6.30 to −1.95)	<0.001	Heavy drinking (yes vs. no)	−3.40 (−5.51 to −1.28)	0.002
			BMI	−0.53 (−0.72 to −0.33)	<0.001
			HTN	3.34 (1.59 to 5.09)	<0.001
			DM	0.89 (−0.70 to 2.49)	0.271
			HC	−2.38 (−3.94 to −0.82)	0.003
			CAD	3.09 (0.32 to 5.85)	0.029
			AF	5.84 (3.31 to 8.37)	<0.001
			LFI	−1.64 (−3.94 to 0.66)	0.161
			RFI	4.44 (2.43 to 6.46)	<0.001

*Note*: Values are *β* coefficients (years) with 95% confidence intervals from multivariable linear regression models in the overall cohort (*n* = 930). Model 1 included sex, smoking, and heavy drinking. Model 2 additionally included BMI, HTN, DM, HC, CAD, AF, LFI, and RFI. Heavy drinking: >45 g/day or >300 g/week of ethanol; nonheavy: abstinent or ≤45 g/day and ≤300 g/week.

Abbreviations: AF, atrial fibrillation; BMI, body mass index; CAD, coronary artery disease; DM, diabetes mellitus; HC: hypercholesterolemia; HTN, hypertension; LFI, liver function impairment (GOT > 40 IU/L or GPT >40 IU/L); RFI, renal function impairment (Creatinine > 1.2 mg/dL).

### Relationship of *ALDH2* and drinking with age at stroke onset

**FIGURE 1:**
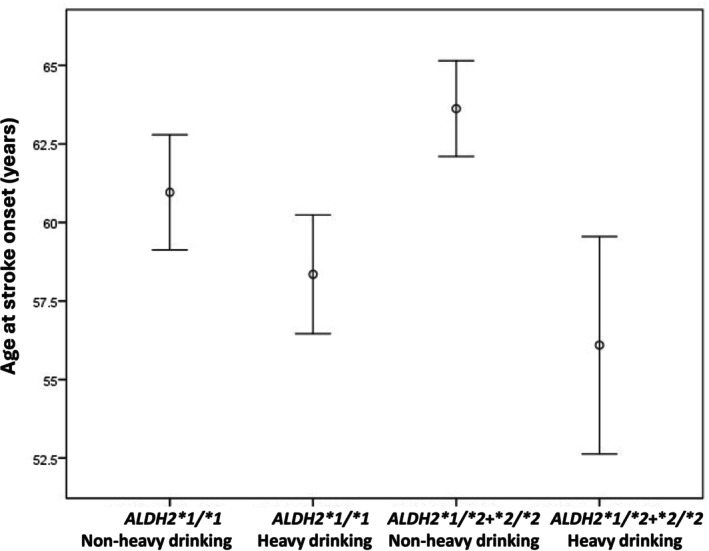
Age at ischemic stroke onset by *ALDH2* genotype and drinking category in men. Mean age at ischemic stroke onset with 95% confidence intervals is shown for the four groups. ALDH2*2 carriers with heavy drinking had the earliest onset, whereas carriers with nonheavy drinking had the latest.

Given the very low prevalence of heavy drinking among women across all *ALDH2* genotypes in our cohort, meaningful stratification by genotype and drinking category was not feasible in women. Therefore, subsequent analyses focused on men, who showed higher alcohol exposure and greater genotype‐related variation. Given the limited number of men with the *2/*2 genotype reporting heavy drinking (*n* = 5), we combined *1/*2 and *2/*2 carriers for statistical analysis. Accordingly, men were stratified into four subgroups according to *ALDH2* genotype and drinking category: *1/*1 carriers with nonheavy drinking, *1/*1 carriers with heavy drinking, *1/*2 + *2/*2 carriers with nonheavy drinking, and *1/*2 + *2/*2 carriers with heavy drinking (Table 3). The age at stroke onset differed significantly across the four groups (*p* < 0.001). Mean age with 95% confidence intervals is shown in Figure 1. Among the four groups, *1/*2 + *2/*2 carriers with heavy drinking had the youngest age at stroke onset (56.1 ± 9.6 years), whereas *1/*2 + *2/*2 carriers with nonheavy drinking had the oldest stroke onset age (63.6 ± 12.3 years). Among *1/*1 carriers, those with heavy drinking had a younger age at stroke onset (58.4 ± 11.0 years) than those with nonheavy drinking (61.0 ± 12.3 years).

**TABLE 3:** Baseline characteristics of male patients with first‐ever ischemic stroke, stratified by *ALDH2* genotype and alcohol consumption status.

	*ALDH2* genotypes and drinking status				*p* value
	*1/*1 nonheavy (*n* = 176)	*1/*1 heavy (*n* = 135)	*1/*2 + *2/*2 nonheavy (*n* = 255)	*1/*2 + *2/*2 heavy (*n* = 32)	
Age, years	61.0 ± 12.3	58.4 ± 11.0	63.6 ± 12.3	56.1 ± 9.6	<0.001^†^
BMI	25.5 ± 3.4	25.6 ± 3.7	24.8 ± 3.5	25.3 ± 3.6	0.112
HTN	128 (72.7)	104 (77.0)	179 (70.2)	19 (59.4)	0.197
DM	77 (43.8)	46 (34.1)	95 (37.3)	11 (34.4)	0.313
HC	99 (56.3)	77 (57.0)	138 (54.1)	16 (50.0)	0.865
CAD	22 (12.5)	7 (5.2)	24 (9.4)	1 (3.1)	0.093
AF	16 (9.1)	12 (8.9)	18 (7.1)	1 (3.1)	0.619
LFI	21 (12.0)	20 (14.8)	36 (14.1)	2 (6.3)	0.560
RFI	36 (20.5)	24 (17.8)	58 (22.7)	5 (15.6)	0.601
Smoking	95 (54.0)	108 (80.0)	154 (60.4)	31 (96.9)	<0.001
TOAST					
Large vessel	54 (30.7)	48 (35.6)	79 (31.0)	6 (18.8)	0.794
Cardioembolism	15 (8.5)	10 (7.4)	19 (7.5)	1 (3.1)	
Small vessel	60 (34.1)	50 (37.0)	92 (36.1)	15 (46.9)	
Other determined	5 (4.5)	5 (3.7)	14 (5.5)	1 (3.1)	
Undetermined	39 (22.2)	22 (16.3)	51 (20.0)	9 (28.1)	

*Note*: Values for age and BMI are expressed as means ± SD. Numbers in parentheses are percentages. Statistically significant differences were determined using the Chi‐square test for categorical variables and one‐way analysis of variance for continuous variables between different groups. Heavy drinking: >45 g/day or >300 g/week of ethanol; Nonheavy: abstinent or ≤45 g/day and ≤300 g/week.

Abbreviations: AF, atrial fibrillation; BMI, body mass index; CAD, coronary artery disease; DM, diabetes mellitus; HC, hypercholesterolemia; HTN, hypertension; LFI, liver function impairment (GOT > 40 IU/L or GPT > 40 IU/L); RFI, renal function impairment (creatinine >1.2 mg/dL); TOAST, Trial of ORG 10172 in Acute Stroke Treatment.

^†^ Post hoc pairwise comparisons (Bonferroni‐adjusted) showed significantly earlier age at stroke onset in *1/*1, heavy vs. *1/*2 + *2/*2, nonheavy (p < 0.001), and in *1/*2 + *2/*2, heavy vs. *1/*2 + *2/*2, nonheavy (*p* = 0.005). Other comparisons were not statistically significant.

Smoking prevalence also differed significantly among the four groups (*p* < 0.001). The highest smoking rate was observed in *1/*2 + *2/*2 carriers with heavy drinking (96.9%), followed by *1/*1 carriers with heavy drinking (80%). Both nonheavy drinking groups exhibited lower smoking rates, particularly *1/*1 carriers with nonheavy drinking (54%), suggesting a strong association between alcohol consumption and smoking behavior in this cohort. Stroke subtype distributions by TOAST classification were comparable across groups (*p* = 0.794).

### Multivariable regression analysis

**FIGURE 2:**
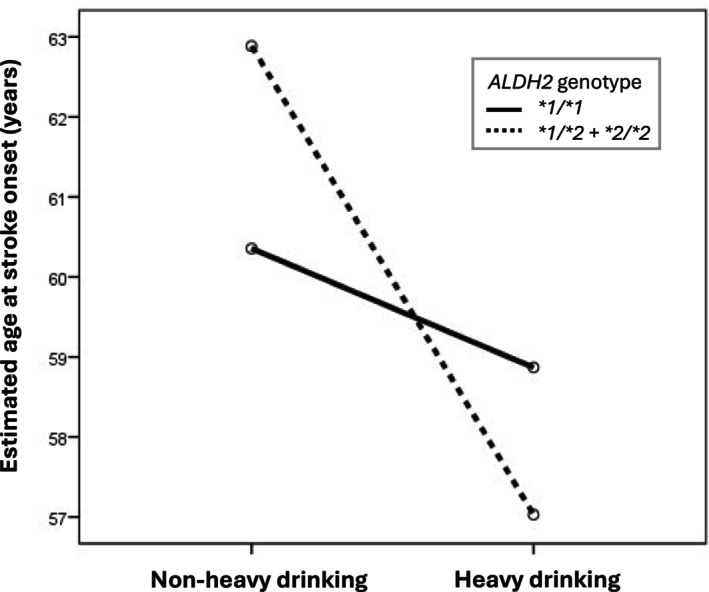
Interaction plot of estimated marginal means of age at stroke onset by *ALDH2* genotype and drinking category in men. Estimated marginal means were derived from a general linear model adjusted for BMI, hypertension, diabetes mellitus, hypercholesterolemia, coronary artery disease, atrial fibrillation, liver dysfunction, renal dysfunction, and smoking. The nonparallel lines indicate a gene–environment interaction between *ALDH2* genotype and drinking category (heavy > 45 g/day or > 300 g/week; nonheavy = abstinent or ≤ 45 g/day and ≤ 300 g/week). Means are shown without standard‐error bars for visual clarity.

A multivariable linear regression, adjusted for smoking status (Table 4), showed that, compared to *1/*1 carriers with nonheavy drinking, *1/*2 + *2/*2 carriers with nonheavy drinking exhibited significantly later age at stroke onset (*β* = 2.67 years, 95% CI: 0.38–4.97, *p* = 0.023), while *1/*2 + *2/*2 carriers with heavy drinking had an earlier onset (*β* = −4.61 years, 95% CI: −9.19 to −0.03, *p* = 0.048). In an alternative model using *1/*2 + *2/*2 carriers with heavy drinking as a reference, *1/*2 + *2/*2 carriers with nonheavy drinking exhibited a significantly later onset (*β* = 7.29 years, 95% CI: 2.84–11.74, *p* = 0.001), suggesting a potentially harmful interaction between the *2 allele and heavy drinking. An interaction model (Model 3) incorporating an ALDH2*2 × heavy drinking term revealed a borderline significant negative interaction effect (*β* = −4.81 years, 95% CI: −9.96 to 0.33, *p* = 0.066), suggesting that heavy drinking might exacerbate the risk of ischemic stroke conferred by the *2 allele, leading to an earlier stroke onset. To further illustrate the joint effect of genotype and alcohol consumption, an interaction plot was generated based on estimated marginal means derived from a general linear model adjusted for all covariates. The resulting plot revealed nonparallel slopes and a crossover pattern, supporting the presence of a gene–environment interaction between ALDH2*2 carriage and heavy drinking in determining age at stroke onset (Figure 2).

**TABLE 4:** Multivariable linear regression analyses of stroke onset age according to *ALDH2* genotype and alcohol consumption.

Model	Reference group	Comparison group	*β* (years), 95% CI	*p* value	Model features
Model 1	*1/*1 Nonheavy	*1/*1 Heavy	−2.47 (−5.20 to 0.26)	0.076	Adjusted for smoking
		*1/*2 + *2/*2 Nonheavy	2.67 (0.38 to 4.97)	0.023	
		*1/*2 + *2/*2 Heavy	−4.61 (−9.19 to −0.03)	0.048	
Model 2	*1/*2 + *2/*2 Heavy	*1/*1 Nonheavy	4.61 (0.03 to 9.19)	0.048	Adjusted for smoking
		*1/*1 Heavy	2.14 (−2.47 to 6.76)	0.362	
		*1/*2 + *2/*2 Nonheavy	7.29 (2.84 to 11.74)	0.001	
Model 3	*1/*1 Nonheavy	ALDH2*2 carrier	2.67 (0.38 to 4.97)	0.023	Interaction model: ALDH2*2 × heavy drinking, adjusted for smoking
		Heavy drinking	−2.47 (−5.20 to 0.26)	0.076	
		ALDH2*2 × heavy drinking	−4.81 (−9.96 to 0.33)	0.066	

*Note*: Values are *β* (years) with 95% CI from multivariable linear regression models; all models are adjusted for smoking. Reference groups are shown in the table. Model 3 additionally includes the interaction term (ALDH2*2 × heavy drinking). Negative *β* indicates younger age at stroke onset. Heavy drinking: >45 g/day or >300 g/week ethanol; nonheavy: abstinent or ≤45 g/day and ≤300 g/week.

## DISCUSSION

This study revealed that Taiwanese men carrying the ALDH2*2 allele who reported heavy drinking had a younger age at first‐ever ischemic stroke than their nonheavy counterparts. These findings suggested that alcohol consumption might modulate the association between *ALDH2* polymorphism and stroke risk. Thus, in this context, alcohol use is not merely a pharmacogenetic behavioral correlate but reflects complex gene–gene and gene–environment interactions. Consistent with previous studies, in our cohort, heavy drinking was often accompanied by cigarette smoking, a major vascular risk factor, which can synergistically increase stroke risk across all *ALDH2* genotypes (Yao et al., 2011).

Previous structural, functional, and cellular studies have demonstrated that the ALDH2*2 variant, which produces a variant polypeptide with a lysine substitution at position 487 (Lys‐487), exerts a dominant‐negative effect by disrupting enzymatic activity and destabilizing the tetrameric structure of the enzyme. In vivo studies further confirmed that hepatic ALDH2 protein levels were reduced by approximately 60%–70% in both heterozygous and homozygous carriers of the variant allele. Enzymatic activity in heterozygotes was about 17% of that observed in individuals with the ALDH2*1/*1 genotype, while activity in homozygotes (ALDH2*2/*2) was too low to be measured (Lai et al., 2014). As a result, individuals with this variant rapidly accumulate acetaldehyde, triggering acute alcohol flush symptoms, such as facial flushing, nausea, tachycardia, and psychosomatic discomfort (Peng et al., 2007). These unpleasant effects often deter the individuals from drinking, which might indirectly lower the risk of stroke (Yao et al., 2011). However, this protective mechanism is not universal. Some ALDH2*2 carriers continue to consume alcohol despite experiencing these adverse effects, placing them at higher risk for several diseases, including esophageal, head and neck, liver, and colorectal cancers, as well as cardiovascular disease (Chang et al., 2017; Chen et al., 2010).

Nonetheless, direct evidence linking the interaction between ALDH2*2 and alcohol consumption to ischemic stroke has been limited—largely because relatively few stroke patients with the ALDH2*2 variant engage in heavy drinking. To address this, our study combined male patients with ALDH2*1/*2 and ALDH2*2/*2 genotypes into a single analysis group and, for the first time, provided supporting evidence that the interaction between this genetic predisposition and heavy alcohol consumption is associated with earlier onset of ischemic stroke (Tables 3 and 4, Figures 1 and 2). This finding is supported by previous studies showing that acetaldehyde accumulation in *ALDH2*‐deficient individuals promotes oxidative stress, endothelial dysfunction, and chronic inflammation, all contributing to cerebrovascular injury (Chen et al., 2008, 2010; Kwon et al., 2014).

Interestingly, across all *ALDH2* genotypes (Table 1), women consistently exhibited a later age at ischemic stroke onset compared with men. This observation aligns with prior studies showing that women typically experience first‐ever strokes at older ages than men, possibly due to the protective effects of estrogen on vascular function and slower accumulation of vascular risk factors earlier in life (Reeves et al., 2008). Moreover, in our cohort, heavy drinking and smoking—both known to accelerate vascular aging—were substantially less common among women, which may further explain the delayed stroke onset observed in female patients.

Sex‐specific effects of *ALDH2* polymorphisms on stroke risk have been consistently observed in previous studies. Some studies have reported stronger or male‐specific associations (Cheng et al., 2018; Sung et al., 2016; Surakka et al., 2023), whereas others have suggested a potential protective effect in women (Hou et al., 2020). A recent meta‐analysis of genome‐wide data across 16 biobanks confirmed a significant association between *ALDH2* variants and ischemic stroke in East Asian men (*p* = 1.67 × 10^−24^) but not in women (*p* = 0.126) (Surakka et al., 2023). These differences might reflect the interaction between genetic susceptibility, alcohol consumption, and hormonal or vascular modulators (Lagranha et al., 2010). In our cohort, heavy alcohol consumption was rare among female patients. Only one woman carrying the ALDH2*1/*2 genotype, who experienced her first‐ever ischemic stroke at the age of 70, reported a history of heavy alcohol consumption. This markedly limited our ability to examine gene–alcohol interactions in female patients. Further, Table 2 showed that male sex, smoking, and heavy drinking were independently associated with earlier stroke onset, with all these factors biologically linked to vascular injury and cumulative risk (Appelros et al., 2009; Shah & Cole, 2010). Together, these findings emphasize that the increased risk of ischemic stroke in individuals with *ALDH2* polymorphisms is not only solely attributable to genetic predisposition but also significantly influenced by environmental factors such as alcohol consumption and smoking.

Although the formal *ALDH2*–alcohol interaction term did not reach statistical significance in our model (Table 4, Model 3, *β* = −4.81 years, 95% CI: −9.96–0.33, *p* = 0.066)—possibly due to the small number of ALDH2*2 carriers with heavy drinking, subgroup analyses revealed consistent and biologically plausible trends. ALDH2*2 carriers with nonheavy drinking exhibited the latest age at stroke onset, followed by *1/*1 carriers with nonheavy drinking. In contrast, ALDH2*2 carriers with heavy drinking exhibited the earliest stroke onset.

Notably, our findings contribute to a growing body of research suggesting that genetic variants may not only influence stroke risk but also affect the age at stroke onset. Previous studies, such as those examining *NINJ2* or *APOE* polymorphisms (Kim et al., 2012; Lagging et al., 2019), have linked specific alleles with earlier or later stroke presentation—even in the absence of traditional vascular risk factors. These findings suggest that genetic factors might influence stroke phenotypes that might lead to long‐term cerebrovascular resilience or vulnerability.

The delayed onset observed among ALDH2*2 carriers with nonheavy drinking suggests the possibility of a context‐dependent protective effect. Individuals with ALDH2 deficiency—who experience unpleasant alcohol‐related symptoms (Peng et al., 2007, 2014)—might engage in healthier behaviors (e.g., reduced alcohol and tobacco use), resulting in decreased cumulative vascular burden. Alternatively, a lack of alcohol exposure might prevent the development of acetaldehyde‐induced oxidative stress, thereby mitigating endothelial damage (Chen et al., 2010). This behavioral‐genetic feedback loop warrants further investigation.

Additionally, *ALDH2* polymorphisms might influence stroke risk and progression via mechanisms beyond alcohol metabolism. ALDH2 plays a key role in detoxifying lipid peroxidation products, such as 4‐hydroxy‐2‐nonenal, preserving mitochondrial integrity, modulating autophagy, and promoting neurovascular repair (Chen et al., 2014). Previous studies have shown that ALDH2 activation can reduce infarct volume and alleviate ischemia‐induced neuronal apoptosis by suppressing the JNK/caspase‐3 signaling pathway and improving mitochondrial membrane potential (Guo et al., 2013; Xia et al., 2020). These findings suggest that ALDH2*2 carriers may be predisposed to earlier strokes due to both alcohol‐induced vascular injury and inherent impairments in mitochondrial defense and neuroprotection.

From a clinical standpoint, our results highlight the importance of considering age at stroke onset as a meaningful endpoint in genetic and epidemiologic studies. While most large‐scale stroke genetics studies focus on disease incidence, the timing of first‐ever stroke might better reflect the interplay between inherited susceptibility and lifetime exposure to modifiable risk factors. Earlier stroke onset often correlates with worse long‐term outcomes, greater economic burden, and disrupted family and occupational roles (Khan et al., 2021). Thus, identifying subgroups at high risk of early‐onset stroke, such as ALDH2*2 carriers with heavy drinking, could inform targeted prevention strategies.

The strength of the current study lies in its relatively large sample size and detailed clinical and genetic data, enabling a stratified analysis of gene–behavior interactions. Given the high prevalence of the ALDH2*2 genotype in East Asians, our findings might be useful for the stroke prevention strategies tailored to this population. Nonetheless, our study had some limitations. First, alcohol consumption was assessed via self‐report or clinical documentation, which might lead to recall or reporting bias. Moreover, alcohol exposure was analyzed categorically rather than as a continuous dose, precluding formal dose–response assessment. In addition, drinking and smoking statuses were based on current behaviors, without detailed quantification of lifetime exposure or duration since cessation. This limitation may have led to an underestimation of the cumulative effects of past drinking or smoking on stroke onset age. Second, though stroke subtypes were analyzed, we did not assess stroke severity or functional outcomes, such as the modified Rankin Scale (mRS). However, our previous study found no association between *ALDH2* genotypes and mRS (Sung et al., 2016), supporting our decision to focus on stroke onset age. Third, due to the cross‐sectional design of our study, causal inference remains limited. Future prospective studies incorporating standardized behavioral data and vascular biomarkers are needed to further explore and validate the mechanisms underlying these gene–environment interactions.

## CONCLUSION

Our study demonstrated that, in a Taiwanese cohort of men with ischemic stroke, carriers of the ALDH2*2 variants who reported heavy drinking had a younger age at stroke onset, suggesting a harmful gene–environment interaction. Conversely, ALDH2*2 carriers with nonheavy drinking exhibited a later onset. These findings highlight the clinical importance of incorporating *ALDH2* genotyping and alcohol consumption assessments into stroke prevention strategies, particularly for East Asian populations.

## FUNDING INFORMATION

Tri‐Service General Hospital (TSGH‐C103‐085, TSGH‐C104‐085, TSGH‐B‐110011, TSGH‐B‐111017, and TSGH‐D‐110049), Taipei Veterans General Hospital, Hsinchu Branch (VHCT‐RD‐2016‐8), Ministry of Science and Technology (MOST 103‐2314‐B‐016‐012 and MOST 104‐2314‐B‐016‐013), and Academia Sinica (grant numbers 40‐05‐GMM and AS‐GC‐110‐MD02).

## CONFLICT OF INTEREST STATEMENT

The authors declare no conflicts of interest.

## Data Availability

The data that support the findings of this study are available on request from the corresponding author. The data are not publicly available due to privacy or ethical restrictions.
